# NAFLD and Diabetes: Two Sides of the Same Coin? Rationale for Gene-Based Personalized NAFLD Treatment

**DOI:** 10.3389/fphar.2019.00877

**Published:** 2019-08-06

**Authors:** Ming-Feng Xia, Hua Bian, Xin Gao

**Affiliations:** ^1^Department of Endocrinology and Metabolism, Zhongshan Hospital, Fudan University, Shanghai, China; ^2^Fudan Institute for Metabolic Diseases, Fudan University, Shanghai, China

**Keywords:** non-alcoholic fatty liver disease, diabetes, gene variant, pathogenesis, personalized treatment

## Abstract

The prevalence of non-alcoholic fatty liver disease (NAFLD) has been increasing rapidly and at the forefront of worldwide concern. Characterized by excessive fat accumulation in the liver, NAFLD regularly coexists with metabolic disorders, including type 2 diabetes, obesity, and cardiovascular disease. It has been well established that the presence of NAFLD increases the incidence of type 2 diabetes, while diabetes aggravates NAFLD to more severe forms of steatohepatitis, cirrhosis, and hepatocellular carcinoma. However, recent progress on the genotype/phenotype relationships in NAFLD patients indicates the development of NAFLD with a relative conservation of glucose metabolism in individuals with specific gene variants, such as the patatin-like phospholipase domain-containing 3 (PNPLA3) and transmembrane 6 superfamily member 2 protein (TM6SF2) variants. This review will focus on the clinical and pathophysiological connections between NAFLD and type 2 diabetes and will also discuss a disproportionate progression of NAFLD and diabetes, and the different responses to lifestyle and drug intervention in NAFLD patients with specific gene variants that may give insight into personalized treatment for NAFLD.

## Introduction

Over the last century, dramatic changes moving toward a sedentary lifestyle, and a high-fat and high-sugar diet, have radically affected human metabolic health status. Chronic metabolic diseases, such as obesity, type 2 diabetes (T2D), and non-alcoholic fatty liver disease (NAFLD), have been increasing at an alarming rate globally in both developed and developing countries (GBD, 2016). The global prevalence of NAFLD is estimated to be 24% at present, with the highest rates in South America (31%) and the Middle East (32%), followed by Asia (27%), the USA (24%), and Europe (23%) (Younossi et al., 2019). According to a global report on diabetes by the World Health Organization (WHO) (WHO, 2016), the prevalence of diabetes is estimated to be 8.5% of the global population or 422 million individuals in 2014. Among diabetic patients, 70−80% have NAFLD (Targher et al., 2007; Williamson et al., 2011). Usually, NAFLD and T2D coexist and act synergistically to drive adverse outcomes in clinical practice. The presence of NAFLD increases the incidence of T2D and accelerates the development of complications in the latter (Shibata et al., 2007; Targher et al., 2007; Targher et al., 2008; Adams et al., 2009;Park et al., 2013). Meanwhile, the presence of T2D increases the likelihood of progression of NAFLD to the more severe forms of liver disorders, such as non-alcoholic steatohepatitis (NASH), cirrhosis, and hepatocellular carcinoma (Adams et al., 2005; Wang et al., 2012).

However, NAFLD is a heterogeneous disease that is influenced by multiple factors, including age, gender, ethnicity, genetic predisposition, and metabolic status (Younossi, 2019). A proportion of individuals develops NAFLD in the absence of obesity and insulin resistance (Younes and Bugianesi, 2019). Recent studies have found that several gene variants in the patatin-like phospholipase domain-containing 3 (PNPLA3) (Romeo et al., 2008), transmembrane 6 superfamily member 2 protein (TM6SF2) (Lallukka and Yki-Järvinen, 2016), glucokinase regulatory protein (GCKR), protein phosphatase 1 regulatory subunit 3B (PPP1R3B), neurocan (NCAN), lysophospholipase-like 1 (LYPLAL1) (Speliotes et al., 2011), and membrane-bound O-acyltransferase domain-containing 7 (MBOAT7) (Mancina et al., 2016) significantly increase the risk of NAFLD. Among them, several gene variants are associated with disproportionate increase in the risks of NAFLD and diabetes. For example, PNPLA3 rs738409 GG gene variant carriers have 73% more liver fat than non-carriers (Sookoian and Pirola, 2011), but are not more likely to have T2D according to the NASH CRN database (Speliotes et al., 2010) and Shanghai Changfeng Study (Xia et al., 2016); only a small increase in the risk of T2D was observed in 100,323 people from a publicly available T2D genome-wide association studies (GWAS) database (OR 1.04 [1.01−1.07], *P* = 0.0045) (Dongiovanni et al., 2018). Moreover, the PNPLA3 gene variant was even associated with a decreased risk of T2D in the NAFLD patients selected from the NASH CRN study (Speliotes et al., 2010) and a Chinese prospective cohort after adjusting for liver fat content and its changes over time (Xia et al., 2019). Another NAFLD-related TM6SF2 rs58542926 C > T gene variant is associated with an average of a 2.1-fold higher risk of NAFLD than non-carriers according to a recent meta-analysis (Pirola and Sookoian, 2015). However, the TM6SF2 gene variant is reported to be accompanied by conserved insulin sensitivity and lower serum triglyceride levels in two Finnish cohorts (Zhou et al., 2015; Sliz et al., 2018), a 20−40% increase in the incidence of T2D in the METSIM and FINRISK studies (Kim et al., 2017), and a small increase in the risk of diabetes in 452,244 individuals from 54 studies (OR 1.07 [1.05−1.10], *P* = 4.8 × 10^−12^) (Mahajan et al., 2018). Other NAFLD-related gene variants, such as LYPLAL1 and MBOAT7, showed no increase in the risk of diabetes (Dongiovanni et al., 2018; Sliz et al., 2018).

This review attempts to demonstrate the clinical and pathophysiological connections between NAFLD and T2D, as well as to explore the disproportionate development of NAFLD and diabetes in individuals with specific genetic variations. We will also review the available evidence regarding the influence of the gene variants on the individual response to lifestyle intervention and drug treatment in patients with NAFLD.

## NAFLD Increases Risks of T2D

The prevalence of diabetes among the NAFLD and NASH patients is estimated to be 22.51% and 43.63%, respectively, which is much higher than the prevalence of diabetes in the general population (8.5%) (WHO, 2016; Younossi et al., 2016). The causal relationship between NAFLD and T2D was initially recognized when high alanine aminotransferase (ALT) was found to predict the development of T2D in Pima Indians (Vozarova et al., 2002). So far, a large number of prospective, population-based cohort studies have demonstrated that the elevation of serum liver enzymes could increase the risk of T2D, independent of other common risk factors (such as diet and lifestyle) in populations from different ethnicities (Goessling et al., 2008; Kunutsor et al., 2013; Ballestri et al., 2016). However, these studies are still limited because most NAFLD patients have normal transaminase levels and complex reasons for liver enzyme elevation. Later, several cohort studies further showed that NAFLD diagnosed by ultrasonography is associated with a 33% to a five-fold increased risk of T2D in different populations with various follow-up periods and severity of NAFLD (Bae et al., 2011; Armstrong et al., 2014). In the last decade, advances in non-invasive methods for measuring liver fat content have enabled studies on the quantitative relationship between NAFLD and T2D. Based on the liver fat content measured by proton magnetic resonance spectroscopy (^1^H-MRS), Cusi et al. found a clear threshold of the liver fat content of ∼6 ± 2%, after which metabolic disorders such as muscle insulin resistance, hypertriglyceridemia, and hypo-high-density lipoprotein cholesterolemia become fully established (Bril et al., 2017). The Shanghai Changfeng Community Study also found that liver fat content > 10% by a quantitative ultrasound method was associated with increased systemic insulin resistance and diabetes (Xia et al., 2015). Taken together, these findings highlight the importance of NAFLD in the occurrence of diabetes. Indeed, prior to the onset of diabetes, the presence of NAFLD is already associated with insulin resistance and elevated nocturnal blood glucose (Bian et al., 2011). Histological liver steatosis grades are inversely associated with hepatic and skeletal muscle insulin sensitivity measured by a euglycemic hyperinsulinemic clamp with 3-[^3^H]-glucose in non-diabetic individuals (Lomonaco et al., 2012).

Concomitant NAFLD in diabetic patients makes it difficult to achieve good blood glucose control (Afolabi et al., 2018) and can aggravate a series of extra-hepatic complications. It has been reported that NAFLD increases the risk of cardiovascular disease by 1.96 (95% CI 1.4−2.7)-fold^9^, chronic kidney disease by 1.87 (95% CI 1.3−4.1)-fold, and proliferative/laser-treated retinopathy by 1.75 (95% CI 1.1−3.7)-fold^10^ in diabetic patients and the distal symmetric polyneuropathy by 5.32 (95% CI 3.1−9.3)-fold in diabetic patients with type 1 diabetes (Mantovani et al., 2017). In a large-scale prospective study based on the US National Health and Nutrition Examination Survey in 1988−1994, advanced fibrosis defined by a high NAFLD fibrosis score was also highly associated with increased cardiovascular disease mortality (HR 3.46 [1.91−6.25]) (Kim et al., 2013).

**Figure 1:**
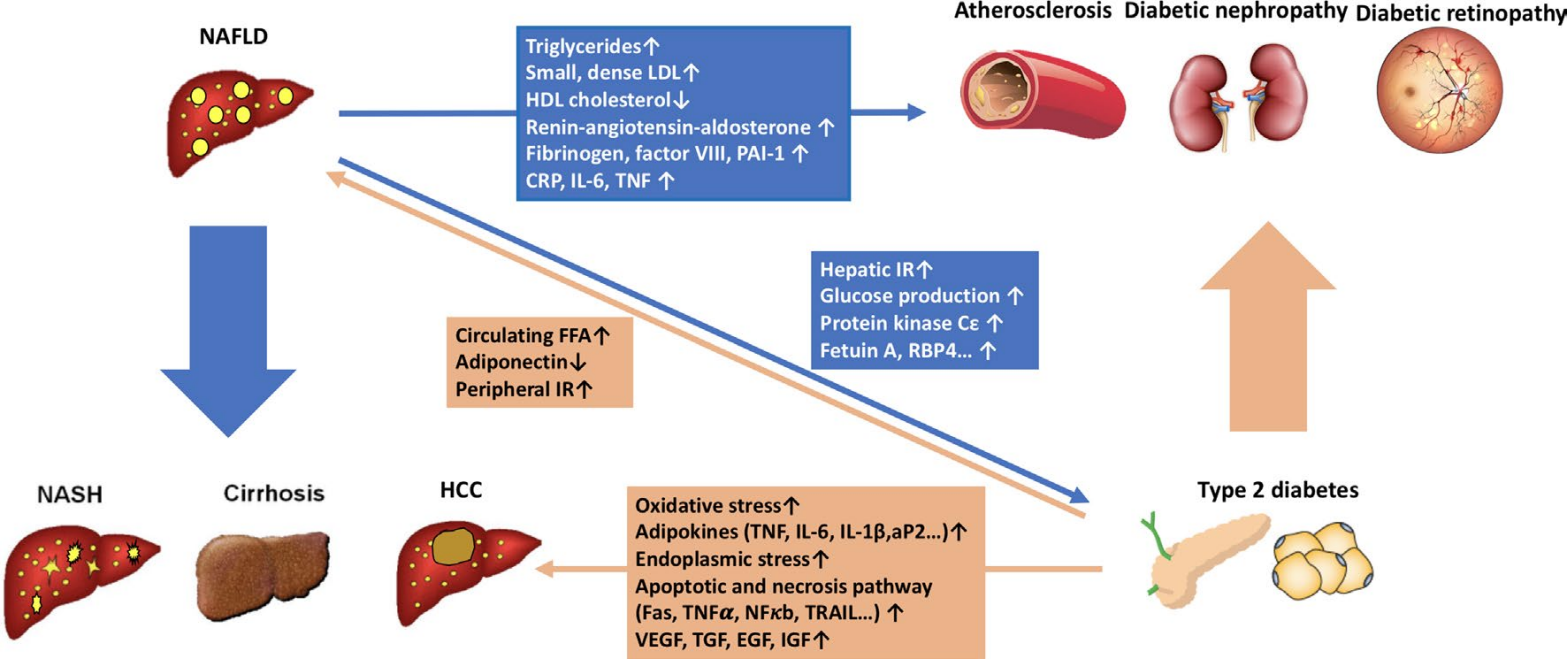
The pathophysiological connections between non-alcoholic fatty liver disease (NAFLD) and type 2 diabetes (T2D). NAFLD contributes to the development of T2D by increasing glucose production in the liver and exacerbating hepatic insulin resistance through the activation of hepatic protein kinase Cε and some liver-secreted proteins with diabetogenic properties, such as fetuin A, fetuin B, RBP4, selenoprotein P, DPP4, and HFREP1. Intrahepatic fat accumulation activates liver inflammation. It further promotes the development of atherogenic dyslipidemia [increased small, dense low-density lipoprotein (LDL) particles, triglycerides, and decreased high-density lipoprotein (HDL) cholesterol] and hypertension (activation of the renin-angiotensin-aldosterone system). It also induces systemic inflammatory status (increased CRP, IL-6, TNF, and reactive oxygen species) and a coagulation mechanism (increased fibrinogen, factor VII, and PAI-1). All the procedures play important roles in the development of diabetic macrovascular and microvascular complications. On the other hand, T2D and systemic insulin resistance promote an increase of free fatty acid flux from peripheral tissues to the liver, leading to the development and progression of NAFLD. Furthermore, T2D drives the progression of NAFLD from simple steatosis to non-alcoholic steatohepatitis (NASH), cirrhosis, and hepatocellular carcinoma through multiple mechanisms, including direct hepatocyte lipotoxicity, hepatocellular oxidative stress due to increased oxidation of free fatty acids, endoplasmic reticulum stress, release of inflammatory cytokines by hepatic Kupffer cells and peripheral adipocytes, hepatocellular apoptosis and necrosis, and hepatocellular regenerative response.

The complex interactions between NAFLD, visceral adiposity, and insulin resistance make it difficult to distinguish the precise mechanisms underlying the increased risk of diabetes in patients with NAFLD. It is likely that expanded and inflamed visceral adipose tissue initiates multiple factors that are potentially involved in the development of insulin resistance and NAFLD, such as free fatty acids and inflammatory adipocytokines (Shoelson et al., 2007). The liver is the main target organ for ectopic fat accumulation, and excessive free fatty acid (FFA) flux into the liver will substantiate insulin resistance by causing lysosomal instability, induction of NF-κB, and activation of TNFα (Feldstein et al., 2004) and cAMP/PKA pathway (Ke et al., 2015), or by activating NLRP3-mediated IL-1β and IL-18 production (Ralston et al., 2017). Diacylglycerol (DAG), an intermediate of liver fat synthesis, also inhibits the liver insulin signaling through activation of protein kinase Cε (PKCε) and c-Jun N-terminal kinase (JNK) (Jornayvaz and Shulman, 2012). As a compensatory mechanism, hepatocytes increase mitochondrial β-oxidation to limit FFA, and the lipid overload will further impair mitochondria antioxidant capacity, cause oxidative stress and mitochondrial leakage, and finally aggravate insulin resistance (Koliaki et al., 2015). Under conditions of hepatic insulin resistance, the *de novo* lipogenesis can be stimulated both by insulin, *via* sterol regulatory element-binding-protein 1c (SREBP-1c) (Tian et al., 2016), and by glucose, *via* carbohydrate response element-binding protein (ChREBP) (Linden et al., 2018). Thus, the interaction between liver steatosis and insulin resistance sets up a vicious cycle to promote the development of both NAFLD and T2D. Several recent studies also demonstrate that liver steatosis alters the secretion of a series of hepatokines with diabetogenic properties, such as fetuin A (Pal et al., 2012), fetuin B (Meex et al., 2015), RBP4 (Norseen et al., 2012), selenoprotein P (Misu et al., 2010), DPP4 (Baumeier et al., 2017), and HFREP1 (Wu et al., 2016). These hepatokines can alter metabolism in liver, muscle, adipose tissue, and pancreas to induce insulin resistance (**Figure 1**).

Liver steatosis promotes atherogenic dyslipidemia [increased small, dense low-density lipoprotein (LDL) particles, triglycerides, and decreased high-density lipoprotein (HDL) cholesterol] (Amor et al., 2017), activates intrahepatic and systemic inflammation [increased C-reactive protein (CRP), interleukin 6 (IL-6), tumor necrosis factor (TNF), intercellular adhesion molecule 1, and P-selectin] (Feldstein et al., 2004; Fricker et al., 2019) and the renin-angiotensin-aldosterone system (RAS) (Oikonomou et al., 2018), and disturbs the coagulation mechanism (increased fibrinogen, factor VII, and PAI-1) (Verrijken et al., 2014). All the NAFLD-related pathogenic mechanisms mentioned above may contribute to the increased risks of diabetic macrovascular and microvascular complications (**Figure 1**).

## T2D Increases the Risk of NAFLD Progression to NASH, Cirrhosis, and Hepatocellular Carcinoma

T2D is one of the strongest clinical predictors of the progression of NAFLD to NASH and cirrhosis (Adams et al., 2005).Current estimates indicate that about 10−20% of NAFLD patients will develop into nonalcoholic steatohepatitis (NASH), thus increasing the risk of liver advanced fibrosis or cirrhosis (Lazo and Clark, 2008). The presence of T2D increases the risk of NASH by two- to three-fold (Portillo-Sanchez et al., 2015). It has been reported that T2D is associated with up to 17.7% advanced liver fibrosis measured by transient elastography (Koehler et al., 2016; Kwok et al., 2016), and 7.1% advanced fibrosis defined by magnetic resonance elastography (MRE) ≥ 3.6 kPa (Doycheva et al., 2016). Studies based on liver histology find that a proportion of patients with T2D exhibits NASH up to 80% and advanced fibrosis to 30−40% (Bazick et al., 2015). There is a great difference in clinical outcomes between NAFLD and NASH patients, and the progression of NAFLD to NASH tremendously increases the annual incidences of liver-specific mortality and hepatic carcinoma from 0.77 and 0.44 per 1,000 person-year, respectively, to 11.77 and 5.29 per 1,000 person-year, respectively, in NAFLD patients (Younossi et al., 2019). A US-based population study in 2010 reported that NAFLD and T2D were the first two common factors present in patients with hepatocellular carcinoma (HCC) (Sanyal et al., 2010), and the presence of diabetes alone can increase the risk of developing HCC two-to-three−-fold (El-Serag et al., 2004). Therefore, the presence of diabetes tremendously drives the progression of NAFLD to NASH, cirrhosis, and even HCC at the final stage. Many patients with NASH developing into HCC exhibit T2D and higher rates of metabolic disorders (such as obesity and hypertension) (Yasui et al., 2011; Aleksandrova et al., 2014). Given the fact that T2D is one of the main conditions closely associated with the progression of NAFLD to NASH, cirrhosis, and HCC, some researchers claim that NAFLD is an overlooked complication of diabetes (Arrese, 2010).

In patients with T2D, liver lipogenesis is elevated (Tian et al., 2016; Linden et al., 2018), and fatty acid oxidation (Schmid et al., 2011) and triglyceride secretion *via* very low-density lipoprotein (VLDL) are decreased (Kamagate and Dong, 2008). Moreover, peripheral insulin resistance increases fatty acid release from adipose tissue (Kim et al., 2017), and the hepatic uptake of fatty acids is also upregulated under the insulin resistance status (Miquilena-Colina et al., 2011). Thus, NAFLD often coexists with T2D. Initially, the mild liver fat accumulation is an adaptive response to metabolic stress against the lipotoxicity of free fatty acids (Donnelly et al., 2005). However, on the background of continuous hepatic free fatty acid influx, various inflammatory pathways are activated with the increasing hepatic intracellular triglycerides (Sharma et al., 2015). In NAFLD patients with T2DM, the progression of NAFLD to NASH, cirrhosis, and even HCC is then driven by multiple insults involving several mechanisms (Buzzetti et al., 2016) (**Figure 1**). The excessive hepatic FFA influx in T2D patients, such as palmitic acid, cholesterol, lysophosphatidylcholine, and ceramides, directly causes lipotoxicity and induces liver inflammation and fibrosis (Tomita et al., 2014; Marra and Svegliati-Baroni, 2018). The oxidation and metabolism of excessive FFAs in the liver further cause oxidative stress (Paradies et al., 2014) and endoplasmic reticulum (ER) stress (Muraki et al., 2017) that trigger hepatocellular damage and apoptosis. In addition, excessive FFAs in the liver, the release of inflammatory mediators from dysfunctional adipose tissue (such as MCP-1, IL-6, and TNFα) (DI Maira et al., 2018), and endotoxins derived from gut (Carnevale et al., 2017) in diabetic patients with NAFLD also activate hepatic Kupffer cells (Kazankov et al., 2019) and release liver inflammatory mediators (IL-1β, TNFα, IL-6) to promote liver injury and inflammation (Yu et al., 2019). Hepatocellular injury further activates the apoptotic and necrotic hepatocyte death pathways (Luedde et al., 2014), and the persistence of this procedure ultimately leads to the activation of hepatic stellate cells, collagen deposition, and hepatic fibrosis (Wree et al., 2014). Recently, it has also been reported that insulin resistance can promote liver fibrosis through induction of lysyl oxidase-like 2 (Loxl2), independent of hepatic stellate cell activity (Dongiovanni et al., 2017). At the same time, the insulin resistance, oxidative stress, ER stress, liver inflammation, and hepatocyte death can also initiate the hepatocellular regenerative mechanism through a series of growth factors and activate multiple oncogenic signaling pathways, such as PI3K/PTEN/Akt, JAK/STAT, NF-kB, mTOR, 4HNE, and NRF-1, which further promote the development of HCC (Noureddin and Rinella, 2015) (**Figure 1**).

## Disproportionate Development of NAFLD and T2D in Specific Gene Variant Carriers

The first evidence for the development of NAFLD in the absence of glucose metabolism disorders was found in a mouse model with overexpression of diacylglycerol acyltransferase 2 (DGAT2), an enzyme catalyzing the final step of hepatic triglyceride biosynthesis from DAG (Monetti et al., 2007). These mice present obvious hepatic steatosis in the absence of any abnormalities in the plasma glucose and insulin resistance levels (Shen et al., 2015). Further, human studies have found that DGAT2 rs1944438 C > T variant carriers have a smaller reduction in liver fat than non-carriers after a lifestyle intervention program, but their changes in insulin sensitivity are not different (Kantartzis et al., 2009). Later, it was recognized that NAFLD-related hepatic insulin resistance is caused by an increase in hepatic DAG content (Jornayvaz and Shulman, 2012). Therefore, individuals carrying the DGAT2 gain-of-function gene variants are associated with an increased risk of NAFLD with disproportionately conserved insulin sensitivity. Moreover, animal studies showed that inhibiting secretion of VLDL from the liver by genetic modification resulted in liver steatosis with conserved insulin sensitivity (Jacobs et al., 2010). Recent advances in GWAS have identified several gene variants that might contribute to the development of NAFLD in a proportion of patients with normal body mass index and a few features of metabolic syndromes, which include the gene variants in PNPLA3, TM6SF2, and MBOAT7 (Romeo et al., 2008; Anstee et al., 2011; Mancina et al., 2016).

**Figure 2:**
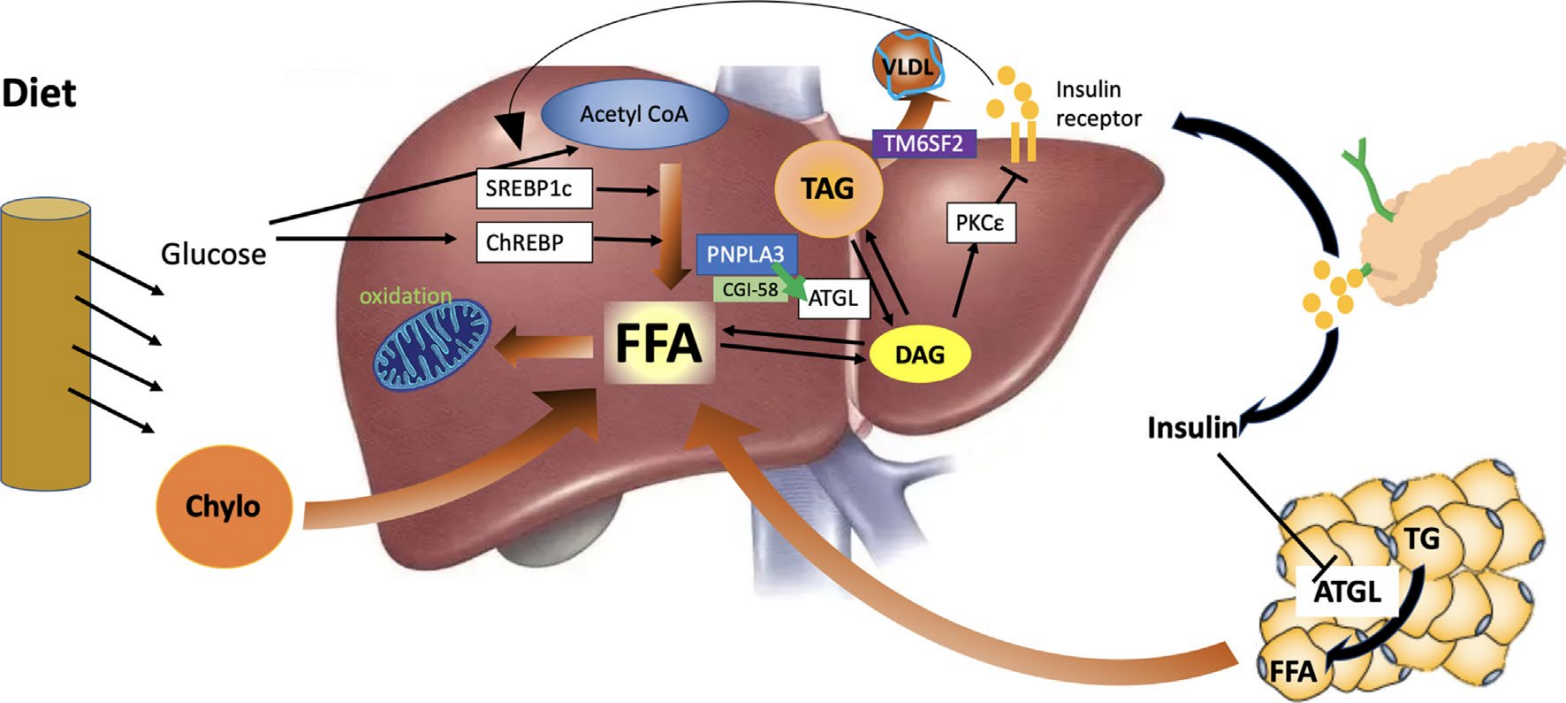
Hepatic lipid metabolism under the condition of insulin resistance and the role of PNPLA3 and TM6SF2. Liver fat is derived from peripheral adipose tissue, *de novo* lipogenesis, and diet intake. In the state of insulin resistance, adipose triglyceride lipase (ATGL) is not fully inhibited by insulin, and free fatty acids are released continuously from the adipose to the liver. Hyperinsulinemia also induces the activity of sterol regulatory element-binding protein 1c (SREBP1c) and *de novo* liver lipid synthesis. The β-oxidation of fatty acid is reduced due to the inhibition of carnitine palmitoyl transferase-1 (CPT-1) by malonyl-coenzyme A generated from *de novo* lipogenesis. PNPLA3 interacts with CGI-58 to regulate the activity of ATGL and the hydrolysis of stored lipids. TM6SF2 functions to facilitate the assembly of very low-density lipoprotein (VLDL). However, mutant PNPLA3 constantly binds with CGI-58, inhibits liver ATGL, and causes liver steatosis and reduced release of insulin-resistance-inducing diacylglycerol (DAG) from lipid droplets. In addition, mutant TM6SF2 can inhibit the mobilization of neutral lipids and assembly of VLDL.

The PNPLA3 gene variant is the first and strongest common variant that is associated with NAFLD (Romeo et al., 2008). The percentage of PNPLA3 gene variant carriers in the population varies from 25 to 70% in different ethnic groups (Romeo et al., 2008; Wagenknecht et al., 2011). PNPLA3 rs738409 GG gene variant carriers have 73% more liver fat, a 3.2-fold higher risk of liver necro-inflammation (Sookoian and Pirola, 2011), and a 1.9-fold higher risk of cirrhosis than PNPLA3 wild-type CC genotype carriers (Shen et al., 2015). PNPLA3 rs738409 CG heterozygous genotype carriers show only a small increase in liver fat content, which is between that of PNPLA3 CC and GG genotype carriers (Romeo et al., 2008; Speliotes et al., 2010; Wagenknecht et al., 2011; Xia et al., 2016). PNPLA3 G allele is also associated with a 1.77-fold risk of HCC (Trépo et al., 2014). However, the fasting or postload glucose and insulin levels do not differ between PNPLA3 gene variant carriers and non-carriers (Petäjä and Yki-Järvinen, 2016), even when insulin resistance level was measured by a hyperinsulinemic euglycemic clamp (Kantartzis et al., 2009), although data from publicly available T2D GWAS database show a small increase in the risk of T2D in PNPLA3 gene variant carriers (Dongiovanni et al., 2018). The PNPLA3 rs738409 C > G variant is also associated with reduced risk of cardiovascular disease and coronary heart disease-associated mortality (Meffert et al., 2017). Therefore, the PNPLA3 rs738409 C > G variant provides an example of a disproportionate progression of NAFLD and diabetes. It is now clear that PNPLA3 is located in lipid droplets (Chamoun et al., 2013), and it may interact with CGI-58 to interfere with adipose triglyceride lipase activity in the liver (Wang et al., 2019), as shown in **Figure 2**. In the PNPLA3 rs738409 C > G variant carriers, mutant PNPLA3 sequesters CGI-58, thus restricting its access to adipose triglyceride lipase and inhibiting the hydrolysis of stored lipids (Wang et al., 2019), which traps both triglyceride and DAG into cellular lipid droplets. It has also been reported that the profile of DAG species in PNPLA3^I148M^ hepatocytes is different from that of PNPLA3^wt^ hepatocytes (Ruhanen et al., 2014), and an unaltered proportion of DAG (FA18:1) in PNPLA3 rs738409 C > G variant carriers with fatty liver may correlate with their conserved insulin sensitivity (Huang et al., 2011; Franko et al., 2018). The subcellular localization and composition of DAG are important for its ability to mediate hepatic insulin resistance (Cantley et al., 2013; Jelenik et al., 2017). Therefore, PNPLA3 rs738409 C > G variant carriers, with a shift of DAG distribution and composition, would present seemingly paradoxical severe liver steatosis and conserved insulin sensitivity. This mechanism is supported by the phenotype of CGI-58 knockdown mice (Cantley et al., 2013). The shift of liver lipid composition from saturated triglycerides to polyunsaturated triglycerides and a marked reduction in ceramides have been reported to contribute to preserved glucose metabolic status in PNPLA3 gene variant carriers (Luukkonen et al., 2016; Mitsche et al., 2018). Although the PNPLA3 gene variant is associated with a disproportionate development of NAFLD and diabetes, the presence of diabetes in the PNPLA3 gene variant carriers still can amplify the genetic effect to drive the progression of NAFLD (Mitsche et al., 2018). Recent studies on the interaction between genetic and environmental factors on NAFLD show that adiposity or metabolic disorders can significantly amplify the effects of gene variants on NAFLD, from steatosis to hepatic inflammation and cirrhosis (Stender et al., 2017). In fact, the expression of PNPLA3 is directly regulated by the insulin-regulated transcription factor sterol regulatory element-binding protein-1c (SREBP-1c), and pathogenic PNPLA3 mutant products accumulate under conditions of obesity and insulin resistance, thus exacerbating liver steatosis, inflammation, and cirrhosis (Huang et al., 2010).

The association between NAFLD and insulin resistance has been observed to be weakened in individuals with TM6SF2 gene variants (Zhou et al., 2015). The frequency of the TM6SF2 rs58542926 C > T gene variants has been reported to be 6.7% in Asians (Wang et al., 2015) and 7% in European populations (Kozlitina et al., 2014). The TM6SF2 gene variant is associated with increased risks of NAFLD, NASH, and advanced fibrosis independent of age, body mass index (BMI), presence of diabetes, and PNPLA3 genotype status (Liu et al., 2014; Sookoian et al., 2015). Insulin sensitivity, as determined by the homeostasis model assessment for insulin resistance (HOMA-IR) or an oral glucose tolerance test, is not reduced in TM6SF2 gene variant carriers (Zhou et al., 2015), and serum triglyceride and LDL cholesterol concentrations are lower compared with non-carriers (Sliz et al., 2018). Although recent GWAS studies show that the TM6SF2 gene variant is associated with a small increase in the risk of diabetes in 452,244 individuals from 54 studies (OR 1.07 [1.05−1.10], *p* = 4.8 × 10^−12^) (Mahajan et al., 2018), the TM6SF2 gene variant has a much larger effect size on NAFLD (OR 2.13 [1.36−3.30], *p* = 0.0009) than T2D (Pirola and Sookoian, 2015). The TM6SF2 gene variant is also associated with lower LDL cholesterol concentrations and a protection from cardiovascular disease (Liu et al., 2017). TM6SF2 is found to be located in the ER and Golgi complex and functions to mobilize neutral lipids for VLDL assembly, and the lipids accumulate in lipid droplets in its absence (Smagris et al., 2016) (**Figure 2**). Consistent with the animal models with inhibition of VLDL secretion, the TM6SF2 gene variant carriers present liver steatosis with no disorders of glucose metabolism (Jacobs et al., 2010), which may be related to the deficiency of polyunsaturated phosphatidylcholines and excess polyunsaturated FFA in the liver of TM6SF2 gene variant carriers (Luukkonen et al., 2017).

Further evidence for NAFLD with a relative conservation of glucose metabolism can also be found in a small number of families with inherited gene mutations, such as familial hypobetalipoproteinemia (Amaro et al., 2010), lysosomal acid lipase deficiency (Reiner et al., 2014), adipose triglyceride lipase (ATGL) (Stefan et al., 2011), and gene mutations related to fatty acid oxidation (such as medium-chain acyl-CoA dehydrogenase deficiency and carnitine palmitoyl transferase-1) (Sun and Lazar, 2013).

## Future of the NAFLD Treatment: Gene-Based Personalization

For the majority of NAFLD patients, NAFLD and T2D/insulin resistance share common pathophysiological factors and coexist with each other, so several current therapies targeted at insulin resistance also demonstrate efficacy in treating NAFLD (Musso et al., 2012; Gao and Fan, 2013). Insulin sensitizers (such as metformin and thiazolidinediones) are one group of effective antidiabetic medication and have been proven to be effective in treating NAFLD in diabetic patients. Metformin is a first-line antidiabetic drug that improves both hepatic and peripheral insulin resistances. Although mounting evidence shows that metformin treatment does not consistently reduce fat content or inflammatory grades in NASH (Said and Akhter, 2017), its use in patients with T2D results in a 50% reduction in HCC incidence (Singh et al., 2013). Thiazolidinediones are peroxisome proliferator-activated receptor gamma (PPARγ) agonists and improve insulin resistance mainly by stimulating adipocyte differentiation (Phielix et al., 2011). Studies on the effect of pioglitazone show a reduction of liver aminotransferase level and an improvement on liver histological steatosis and inflammation in NAFLD patients with T2D (Cusi et al., 2016). Other novel treatments, such as glucagon-like peptide 1 (GLP-1) receptor agonists (Armstrong et al., 2016) and bariatric surgery (Lassailly et al., 2015), also show promising results in decreasing body weight and insulin resistance and improving NAFLD histological changes, but their effects on NAFLD still need to be further demonstrated in more populations with longer treatment periods. In patients with concomitant NAFLD and T2D, reduction in liver fat content can also help them achieve better blood glucose control and improve the long-term response to lifestyle intervention (Schmid et al., 2017).

NAFLD patients with several specific gene variants feature NAFLD with a relative conservation of glucose metabolism, but there are still no formal recommendations for this type of NAFLD. Several preliminary clinical trials have indicated that NAFLD patients with specific gene variants respond differently to lifestyle and drug intervention. To take the PNPLA3 gene variant carriers as an example, a 6-day short-term hypocaloric low-carbohydrate diet led to a greater decrease in liver fat content in Finnish individuals with the PNPLA3 GG genotype than those with the PNPLA3 CC genotype (45% *vs*. 18%) (Sevastianova et al., 2011). In a 12-month lifestyle intervention program with >10% body weight reduction, PNPLA3 GG homozygotes showed a significantly greater reduction in liver fat content than PNPLA3 CG heterozygotes and CC homozygotes (Shen et al., 2015). In severely obese patients with NAFLD, the PNPLA3 G carriers had significantly greater improvement in hepatic steatosis 1 year after bariatric surgery with an average weight loss of 40 kg (Krawczyk et al., 2016). However, the Wessex Evaluation of fatty Liver and Cardiovascular markers in NAFLD with OMacor thErapy (WELCOME) trial showed the opposite result that liver fat content was decreased due to DHA+EPA treatment only in PNPLA3 CC and CG, but not GG genotype carriers (Scorletti et al., 2015). A recent EFFECT-II study investigated the effects of dapagliflozin and omega-3 carboxylic acid on liver steatosis and, interestingly, found that compared with PNPLA3 wild-type genotype carriers, PNPLA3 gene variant carriers shower a smaller reduction in liver fat content in the dapagliflozin treatment group, similar liver fat reduction in the omega-3 carboxylic acid treatment group, and larger reduction in liver fat content in the combination treatment group (Eriksson et al., 2018). Many clinical trials are still ongoing to evaluate the difference among the NAFLD patients with different gene variants in their response to different NAFLD interventions. Although it is still an emerging new frontier to study gene variation and the efficacy of NAFLD treatment, all the current evidence indicates that a personalized treatment based on genetic classification is necessary.

## Conclusion

It is clear that there is a complex bidirectional relationship between the progression of NAFLD and the development of T2D, and their interaction could result in an increase in both hepatic and diabetic mortalities in patients with concomitant NAFLD and T2D. For NAFLD patients with T2D, some currently available therapies targeting insulin resistance might be the best choice to improve both hepatic and metabolic outcomes. For NAFLD patients with specific gene variant and a disproportionate conservation of glucose metabolism, preliminary data indicate a different response to current NAFLD interventions. Therefore, more studies on the treatment of NAFLD in specific gene variant carriers are urgently needed, and a testing of NAFLD-related gene variants may be helpful to guide personalized treatment in the near future.

## Author Contributions

M-FX, HB and XG contributed conception of the review. M-FX wrote the first draft of the manuscript; HB and XG wrote sections of the manuscript and revised the whole manuscript. All authors contributed to manuscript revision, read and approved the submitted version.

## Funding

This work was supported by the National Key Research Program of China (grant number 2012CB524906 to XG, 2017YFC1309800, 2017YFC1309801 to HB, and 2017YFC1309804 to M-FX), the Shanghai Municipal Science and Technology Committee (grant numbers 16411954800 to XG), the Shanghai Health and Family Planning Commission Foundation (grant numbers 15GWZK0801 to XG), the Shanghai Municipal Science and Technology Major Project (grant number 2017SHZDZX01 to XG), and key basic research grants from Science and Technology Commission of Shanghai Municipality (grant number 16JC1400500 to XG).

## Conflict of Interest Statement

The authors declare that the research was conducted in the absence of any commercial or financial relationships that could be construed as a potential conflict of interest.
